# Application of an Artificial Intelligence Algorithm to Prognostically Stratify Grade II Gliomas

**DOI:** 10.3390/cancers12010050

**Published:** 2019-12-22

**Authors:** Daniela Cesselli, Tamara Ius, Miriam Isola, Fabio Del Ben, Giacomo Da Col, Michela Bulfoni, Matteo Turetta, Enrico Pegolo, Stefania Marzinotto, Cathryn Anne Scott, Laura Mariuzzi, Carla Di Loreto, Antonio Paolo Beltrami, Miran Skrap

**Affiliations:** 1 Department of Medicine, University of Udine, 33100 Udine, Italy; miriam.isola@uniud.it (M.I.); delben.fabio@spes.uniud.it (F.D.B.); cathryn.scott@uniud.it (C.A.S.); laura.mariuzzi@uniud.it (L.M.); carla.diloreto@uniud.it (C.D.L.); 2 Department of Pathology, University Hospital of Udine, 33100 Udine, Italy; michela.bulfoni@uniud.it (M.B.); enrico.pegolo@asuiud.sanita.fvg.it (E.P.); stefania.marzinotto@asuiud.sanita.fvg.it (S.M.); 3 Department of Neurosurgery, University Hospital of Udine, 33100 Udine, Italy; tamara.ius@asuiud.sanita.fvg.it (T.I.); skrap@asuiud.sanita.fvg.it (M.S.); 4 Immunopathology and Cancer Biomarkers, Centro di Riferimento Oncologico di Aviano (CRO) IRCCS, 33081 Aviano (PN), Italy; matteosalotto@gmail.com; 5 SISSA (Scuola Internazionale Superiore di Studi Avanzati), 34136 Trieste, Italy; Giacomo.Da@aau.at

**Keywords:** grade II glioma, prognosis, extent of resection, molecular classification, MRI data, artificial intelligence, decision trees

## Abstract

(1) Background: Recently, it has been shown that the extent of resection (EOR) and molecular classification of low-grade gliomas (LGGs) are endowed with prognostic significance. However, a prognostic stratification of patients able to give specific weight to the single parameters able to predict prognosis is still missing. Here, we adopt classic statistics and an artificial intelligence algorithm to define a multiparametric prognostic stratification of grade II glioma patients. (2) Methods: 241 adults who underwent surgery for a supratentorial LGG were included. Clinical, neuroradiological, surgical, histopathological and molecular data were assessed for their ability to predict overall survival (OS), progression-free survival (PFS), and malignant progression-free survival (MPFS). Finally, a decision-tree algorithm was employed to stratify patients. (3) Results: Classic statistics confirmed EOR, pre-operative- and post-operative tumor volumes, Ki67, and the molecular classification as independent predictors of OS, PFS, and MPFS. The decision tree approach provided an algorithm capable of identifying prognostic factors and defining both the cut-off levels and the hierarchy to be used in order to delineate specific prognostic classes with high positive predictive value. Key results were the superior role of EOR on that of molecular class, the importance of second surgery, and the role of different prognostic factors within the three molecular classes. (4) Conclusions: This study proposes a stratification of LGG patients based on the different combinations of clinical, molecular, and imaging data, adopting a supervised non-parametric learning method. If validated in independent case studies, the clinical utility of this innovative stratification approach might be proved.

## 1. Introduction

Diffuse WHO grade II gliomas have a more favorable prognosis than grade III and IV gliomas but represent a clinical challenge due to their clinical heterogeneity [1,2]. In particular, there is lack of consensus on the criteria to be employed to identify candidates to adjuvant chemo- or radiotherapy, since both treatments have relevant side effects, such as a progressive decline in attentional functioning [3]. Hence, a personalized approach to post-surgical clinical management, in terms of the type of adjuvant therapy and follow-up protocols, requires a correct stratification of the patients in terms of risk of recurrence, risk of malignant transformation, and knowledge of the time interval in which the latter occurs.

The extent of surgical resection (EOR) and the molecular classification of LGGs based on *IDH* mutation and 1p/19q chromosome deletion have recently been shown to have prognostic significance. Indeed, several studies have validated the prognostic role of the 2016 WHO classification of brain tumors [4] that integrates crucial findings emerging from keynote genome-wide studies conducted on lower-grade gliomas [5,6,7]. Similarly, the role of EOR in predicting prognosis and preventing malignant transformation has been well documented [8,9,10,11,12,13,14,15]. Van den Bent’s group has confirmed in a series of 228 LGGs that both EOR and tumor molecular class exert independent prognostic roles [16]; furthermore, especially in the *IDH* mutated astrocytomas, a post-operative volume of 0.5–1 cm^3^ was sufficient to affect prognosis negatively, suggesting the possible importance of a second surgery in these patients. This paper represents an attempt to relate the risk of reduced OS on the basis of the degree of residual tumor mass and the molecular class [16].

Indeed, what is still missing is an algorithm that is able not only to define the factors that impact patient prognosis but to give them hierarchical importance and to define clear cut-off values that could allow clinicians to easily stratify patients on a multiparametric basis.

Therefore, goal of the present study is to use molecular and clinical data to predict overall survival (OS), progression-free survival (PFS) and malignant progression-free survival (MPFS) on one of the largest monocentric series of LGGs available (241 patients) by means of an artificial intelligence algorithm.

There are a number of automatic computer-based systems able to predict the risk of recurrence in a given patient. These approaches can be of great aid in visualizing and interpreting complex datasets [17]. One of these is the algorithmic approach by means of decision trees (DT), which is a supervised non-parametric learning method used for classification [17]. The algorithm is able to detect automatically the most relevant parameters and their optimal cut-offs and divide the population into homogenous subgroups with respect to a target variable, such as time to recurrence, thus allowing clinicians to stratify patients on a multiparametric basis by identifying those factors that affect their prognosis, each with its hierarchical level, on the basis of a clear cut-off value [17].

## 2. Results

### 2.1. Patients Included in the Study

Between 2000 and 2017, 345 patients underwent surgical resection for a grade II glioma (LGG) at the Hospital of Udine. A total of 104 patients were excluded: 40 because molecular data were unobtainable, 15 had undergone only a biopsy, in 15 follow-up data were unavailable, and 34 because tumor diagnosis was incidental. We have shown that these cases have an excellent prognosis regardless of molecular type [18]. The clinical data of the 241 patients included in this study are summarized in Table 1 and the pathological and molecular features in Table 2.

A slight prevalence for the male sex was seen (59.8% vs. 40.2%) and median age was of 39 years (range 19–75 years). The median KPS at surgery was 100% (range 80–100%). The tumors were equally distributed between left and right hemispheres; the frontal, insular, parietal, and temporal lobes were involved, respectively, in 40.2%, 29.9%, 13.7%, and 17.2% of cases.

The median pre-operative volume of the tumors, calculated in T2 sequences, was 44 cm^3^ (range 6–260 cm^3^), while the median difference in pre-operative volumes, as measured between T1 and the T2 sequences (ΔVT2T1), was 13 cm^3^ (0–95 cm^3^). ΔVT2T1 is considered an index of tumor infiltration [9,11,19]. The median EOR was 86% (range 28–100%) and the median post-operative volume 7 cm^3^ (range 0–125 cm^3^). At progression and/or malignant transformation, 73.4% of LGG patients underwent radiotherapy, 75.1% chemotherapy and 37.8% second surgery.

The distribution of LGGs according to the 2016 WHO classification of brain tumors [4] was diffuse astrocytomas, IDH-mutant (astrocytomas IDHmt), oligodendrogliomas, IDH-mutant, and 1p/19q-codeleted (oligodendrogliomas), and diffuse astrocytoma, IDH-wildtype (astrocytomas IDHwt) in 56.8%, 32.0%, and 11.2%, of cases, respectively. Discrepancies between the 2007 WHO [20] and the 2016 WHO [4] classifications of brain tumors were detected. Specifically, while 7.3% of astrocytomas were recognized as oligodendrogliomas, oligoastrocytomas were re-classified as astrocytomas IDHwt, astrocytomas IDHmt and oligodendrogliomas in 3.1%, 33.8%, and 63.1% of cases, respectively.

Among *IDH* mutated cases, 88.1% and 11.9% of LGGs presented mutations in *IDH*1 and *IDH*2 genes, respectively. R132H was the most frequent *IDH* mutation (76.1% of LGG). Overall median Ki67 expression was 4% (range 1–22%). The promoter of MGMT was un-methylated in 12.5% of LGGs. Methylation was assessed by analyzing the methylation levels of 4 CpG islands; the promoter was considered methylated when the average methylation level was at least 9%. In LGGs, the median average methylation level of the MGMT promoter was 25.5% (range 2.5–80.75).

When comparing the clinical and neuroradiological features of the different molecular classes, some significant differences were apparent (Table 1). Age differed significantly in the three classes (*p* = 0.0001), patients with oligodendrogliomas being younger than those with astrocytomas IDHwt and older than those with astrocytomas IDHmt (median age 41 vs. 51 vs. 36 years, respectively). Tumor location significantly differed in the three molecular classes, with astrocytomas IDHwt rarely occurring in the frontal lobe (*p* = 0.006).

No significant differences were detected in pre-operative volumes among the three classes of LGGs. Conversely, both EOR (*p* = 0.008) and post-operative volumes (*p* = 0.027) differed significantly among the three groups. The median EOR was larger in oligodendrogliomas than in astrocytomas IDHwt (87% vs. 69%; *p* = 0.0046) and tended to be also larger in astrocytomas IDHmt than in astrocytomas IDHwt (86% vs. 69%; *p* = 0.057). This was confirmed by considering the EOR as a categoric variable: in this way, more than half of astrocytomas IDHwt had an EOR <70% (48.3% of cases vs. 16.8% and 10.4% of astrocytomas IDHmt and oligodendrogliomas, respectively; *p* < 0.0001). Accordingly, the median post-operative volumes differed significantly between the three molecular classes (*p* = 0.003), the residual tumor of oligodendrogliomas being smaller than that of both astrocytomas IDHwt (*p* = 0.029) and astrocytomas IDHmt (*p* = 0.044). In fact, only 1.3% of oligodendrogliomas presented a post-operative volume >30 cm^3^, versus 12.4% of astrocytomas IDHmt and 25.9% of astrocytomas IDHwt.

Since both EOR and tumor sites significantly differed in the three molecular classes, we wondered whether the EOR could be influenced by a preferential location of the different molecular subtypes for sites surgically treatable with distinctive efficiency. However, no significant differences were detected in the EOR in the different tumor sites (Kruskal–Wallis equality-of-populations rank test: *p* = 0.4168). This suggests that EOR could be affected by tumor biological properties other than the preferential location for a specific brain region.

When comparing pathological and molecular findings with the three molecular classes (Table 2), ATRX down-regulation was mainly restricted to astrocytomas IDHmt (*p* < 0.0001), and the fraction of p53-positive tumors was 90.4% in astrocytomas IDHmt vs. 46.2% in astrocytoma IDHwt and 35.7% in oligodendrogliomas (*p* < 0.0001). Unmethylated tumors were seen among 40% of astrocytomas IDHwt versus 1.4% of oligodendrogliomas and 13.5% of astrocytomas IDHmt (*p* < 0.0001). Accordingly, the average methylation levels of the MGMT promoter was significantly lower in astrocytomas IDHwt (14.3%) versus both astrocytomas IDHmt (24.1%; *p* = 0.030) and oligodendrogliomas (32.4%; *p* = 0.0001). The difference between astrocytomas IDHmt and oligodendrogliomas was also significant (*p* = 0.002).

### 2.2. Molecular Classification, EOR and ΔVT2T1 Independently Predict Patient Prognosis.

#### 2.2.1. Overall Survival

Overall, there were 123 deaths (51%), and the median OS observed was 66 months (range 13–239). The estimated 3-, 5-, and 10- year OS was 84.9%, 72.5%, and 36.8%, respectively.

Factors identified as significantly associated with OS at univariate analysis (*p* < 0.05) were (Table 3): age, KPS, the pre-operative tumor volume, the infiltrative growth index ΔVT2T1, both EOR and the post-operative tumor volume, the expression of Ki67, the presence of mutated *IDH*1 or *IDH*2 genes, the 1p/19q co-deletion, the tumor molecular class, and the methylation status of the MGMT promoter.

Upon multivariate Cox analysis, the independent predictors of OS were post-operative tumor volume, EOR, Ki67 expression, molecular class, KPS, and second surgery (Table 4).

#### 2.2.2. Progression-Free Survival

Evidence of tumor progression was seen in 185 cases (76.8%), and the median PFS was 40 months (range 4–185). The estimated 3-, 5-, and 10-year PFS were 62.6%, 40.4%, and 7.4%, respectively. As shown in Table 3, tumor site, both pre-operative and post-operative tumor volumes, ΔVT2T1, EOR, Ki67 expression, *IDH*1/2 gene mutation, 1p/19q co-deletion, molecular class, and methylation status of the MGMT promoter were associated with PFS at Cox univariate analysis. Upon multivariate Cox analysis, the independent predictors of PFS were the infiltrative growth index ΔVT2T1, Ki67 expression, the molecular class and EOR (Table 4).

#### 2.2.3. Malignant Progression-Free Survival

Anaplastic transformation was observed in 152 cases (63.1%). The median MPFS was 53 months (range 6–239). The estimated 3-, 5-, and 10-year MPFS rates were 75.2%, 57.8%, and 22.4%, respectively. As shown in Table 3, the prognostic factors associated with MPFS upon univariate Cox analyses were the same as those associated with OS with the exception of Ki67 expression. On multivariate Cox analysis, the independent predictors of MPFS were the post-operative tumor volume, the EOR, the infiltrative growth index, the molecular class, and the KPS (Table 4).

#### 2.2.4. Survival Curves

**Figure 1:**
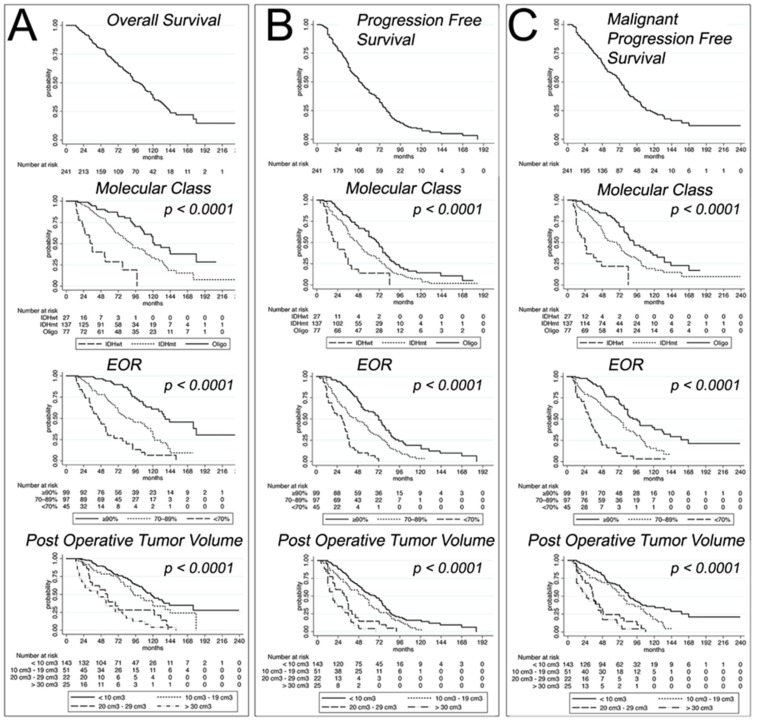
Kaplan–Meyer estimates of OS (**A**), PFS (**B**), and MPFS (**C**) of 241 patients stratified according to molecular class, EOR, and post-operative tumor volume.

Figure 1 shows the OS, PFS, and MPFS survival curves of LGG, stratified according to the molecular class, the EOR, and the post-operative tumor volume.

### 2.3. Adoption of a Decision Tree Approach to Stratify Patients

Finally, we used a classification algorithm based on DT to subdivide the patient population into uniform subgroups in relation to OS, PFS, and MPFS. Specifically, we selected three different thresholds (3, 5, and 10 years) for each subgroup; to avoid the bias of an insufficient follow-up for some patients below the thresholds, we removed those who did not present the studied event.

**Figure 2:**
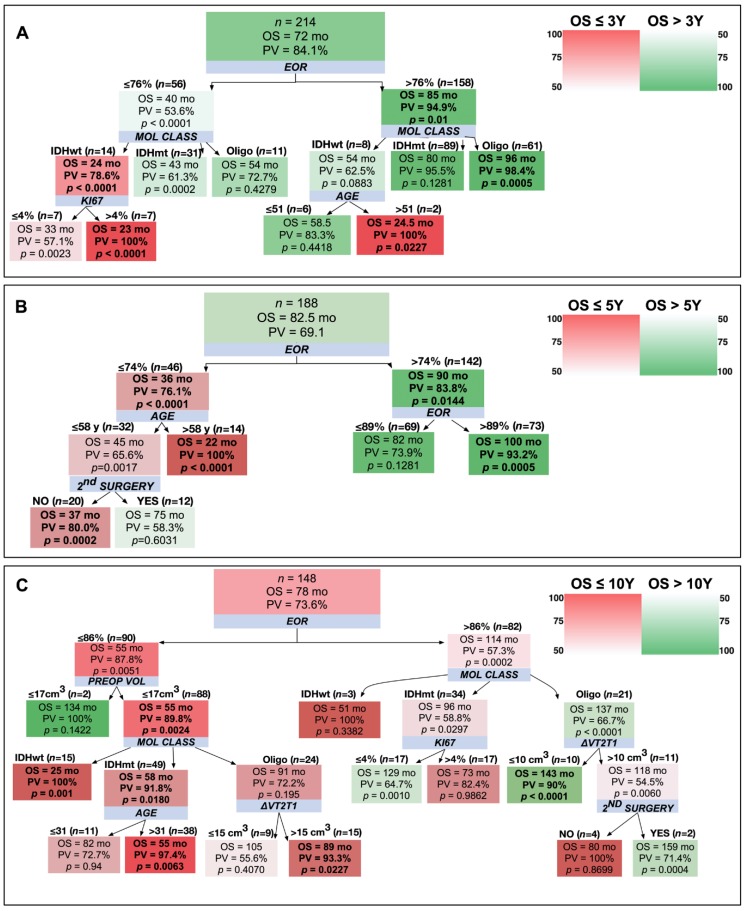
Decision trees (DT) to predict an OS >3-years (**A**), 5-years (**B**), and 10-years (**C**). The shading color of the cells indicates the predictive value (PV) of an OS superior (green) or inferior (red) to 3 (A), 5 (B) or 10 years (C) (see color heat map). The top box of each DT describes the starting population (number, median OS (OS), and PV). Blue boxes show the variables stratifying the population. On top of the detected subgroups are shown the cut-off values and the number of patients satisfying them. Within the branches, boxes show the median OS of the patient subgroup, the PV, and the results of the Mann–Whitney test (*p*-value) comparing the subgroup OS with that of the general population (top box). In bold are indicated the subgroups characterized by a PV >75% and a *p* < 0.05.

First, we defined patients with a particularly poor prognosis (OS <3 years) (Figure 2A and Table 5). The most important parameter was the EOR. In fact, an OS >3 years was observed in 94.9% of patients with an EOR >76% (median OS (mOS) 85 months) vs. 53.6% of those with an EOR ≤76% (mOS 40 months). The molecular class further refined patients’ prognosis. While 95.5% of astrocytomas IDHmt and 98.4% of oligodendroglioma patients with an EOR >76% were still alive after three years, 37.5% of astrocytomas IDHwt patients died within three years. Similarly, the molecular class contributed to stratify the prognosis of patients with an EOR ≤76%. In fact, 78.6% of astrocytoma IDHwt patients died before three years, being those with a Ki67 >4% or an age >51 years particularly at risk. Conversely, 72.7% and 61.3% of oligodendroglioma and IDHmt astrocytoma patients were still alive at the end of the follow-up.

We discriminated patients with an OS >5 years (Figure 2B). EOR was still the most relevant parameter: 83.8% of the patients with EOR >74% survived more than 5 years (mOS 90 months). This fraction rose to 93.2% if the EOR was >89% (mOS 100 months). To stratify the population of long survivors within the heterogeneous population of patients with an EOR comprised between 74% and 89%, Ki67 became a relevant parameter. In particular, 83.3% of patients with Ki67 ≤5% were able to survive beyond 5 years (mOS 88.5 months). In cases with an EOR ≤74%, all the patients older than 58 years showed an OS <5 years (mOS 22 months). The patients that presented a higher chance of surviving more than 5 years had less than 58 years and underwent second surgery (mOS 74.5 months).

Long-time survival with an OS >10 years (Figure 2C) was bound to a good surgery outcome (EOR >86%). For those patients that had an optimal surgery, the molecular class further identified the longest survivors; in fact, whereas no IDHwt astrocytoma patient reached this endpoint, 41.2% and 66.7% of IDHmt astrocytoma and oligodendroglioma patients did. In the oligodendroglioma class, the probability of surviving more than 10 years was further increased by a low infiltrative index (≤18 cm^3^) or by the opportunity of second surgery. In astrocytoma IDHmt, a Ki67% ≤4% further improved the prognosis. Among patients with an EOR<86%, only those with a very small preoperative volume (≤17 cm^3^) were long survivors. Patients with a larger preoperative tumor volume, a molecular class astrocytoma IDHwt, an astrocytoma IDHmt with an age >31 years or an oligodendroglioma with an infiltrative index >15 cm^3^ did not reach 10-year survival.

In addition, we compared the classification accuracy of the chosen approach with two more advanced versions of DT, namely random forest and boosting trees (Table 6). Random forest offered a slightly increased accuracy, at the expense of a largely reduced transparency of the algorithm behavior. ADA boost, on the other hand, did not show a significant improvement over DT alone.

A similar analysis was conducted on PFS and MPFS. As shown in Supplementary Figures S1 and S2, the EOR is still the most relevant parameter, able to stratify the risk of progression. The molecular class is able to play a role mostly in the presence of suboptimal surgery.

Finally, we decided to adopt the same decision tree approach to identify factors influencing the OS of the different molecular groups.

**Figure 3:**
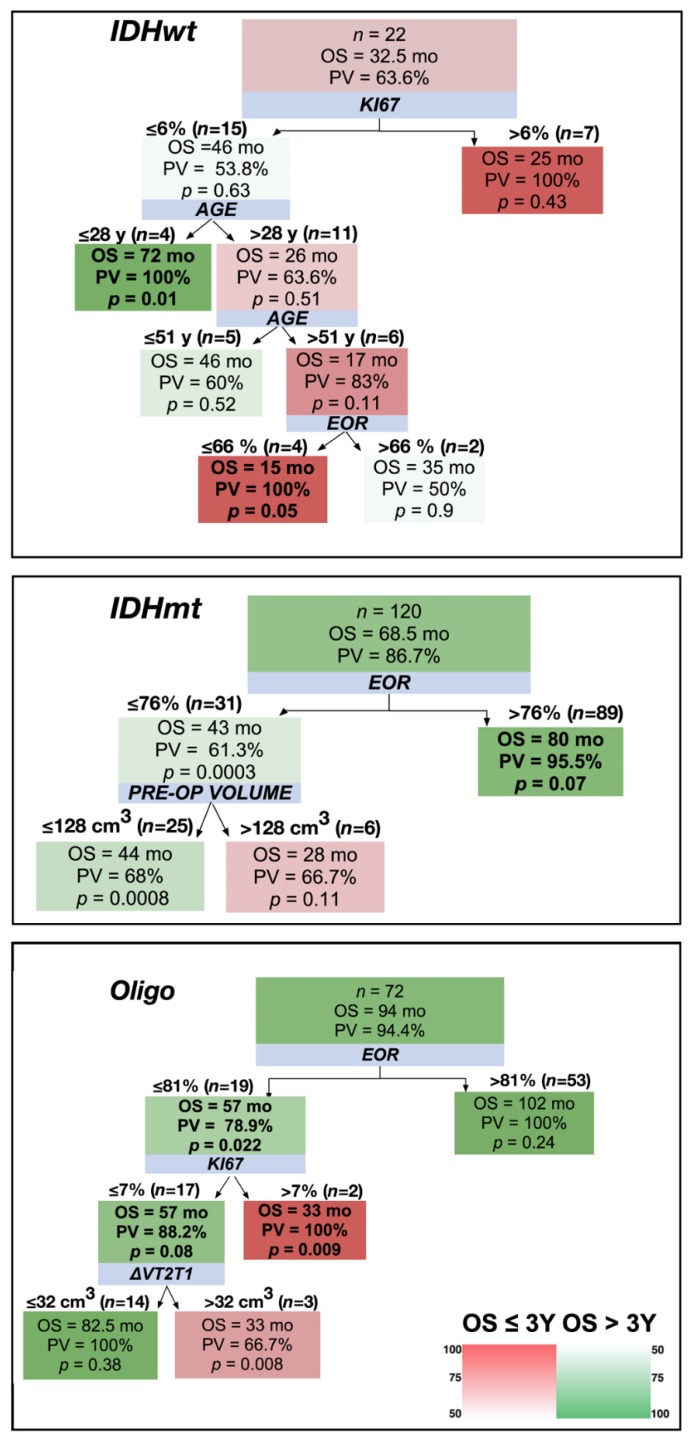
Decision trees applied to the three LGG molecular classes considering 3-year OS as a cut-off. The shading color of the cells indicates the predictive value (PV) of an OS superior (green) or inferior (red) to 3 years (see color heat map). Blue boxes show the variables stratifying the population. On top of the detected subgroups are shown the cut-off values and the number of patients satisfying them. Within the branches, boxes show the median OS of the patient subgroup, the PV, and the results of the Mann–Whitney test (*p*-value) comparing the subgroup OS with that of the general population (top box). In bold are indicated the subgroups characterized by a PV >75% and a *p* < 0.05.

As shown in Figure 3, 63.6% of astrocytomas IDHwt were characterized by an OS <3 years. Short term survivors either had a Ki67 >6% or were older than 28 years, even with a Ki67 ≤6%. Only 13.3% of astrocytomas IDHmt died within 3 years. 25% of these short-term survivors were characterized by an EOR ≤76% and a large pre-operative tumor volume (>128 cm^3^). Oligodendrogliomas survived more than 3 years in 94.4% of cases. The few patients that died showed a Ki67 >7% or a high infiltrative index (ΔVT2T1 >32 cm^3^).

**Figure 4:**
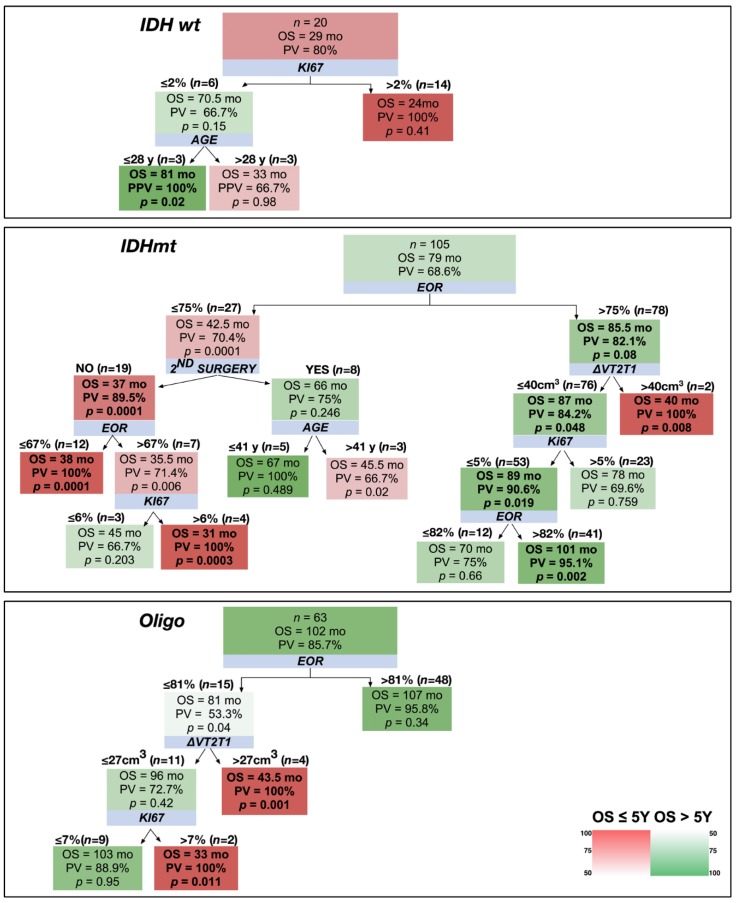
Decision trees applied to the three LGG molecular classes considering 5-year OS as a cut-off. The shading color of the cells indicates the predictive value (PV) of an OS superior (green) or inferior (red) to 5 years (see color heat map). Blue boxes show the variables stratifying the population. On top of the detected subgroups are shown the cut-off values and the number of patients satisfying them. Within the branches, boxes show the median OS of the patient subgroup, the PV, and the results of the Mann–Whitney test (*p*-value) comparing the subgroup OS with that of the general population (top box). In bold are indicated the subgroups characterized by a PV >75% and a *p* < 0.05.

Considering the OS of >5 years cut-off, 100% of astrocytomas IDHwt with a Ki67 of ≤2% and an age of ≤28 years reached this endpoint (Figure 4). In the astrocytomas IDHmt group, an EOR of >75% and a ΔVT2T1 of ≤40 cm^3^ identified patients with an OS of >5 years with a PPV of 84.2%. On the contrary, 70.4% of patients with an EOR of ≤75% died before 5 years. Within this group of patients, those that had a more benign prognosis had been subjected to second surgery or had an age of ≤41 years. Regarding oligodendrogliomas, 85.7% of patients survived more than 5 years. The few patients that did not survive at 5 years had an EOR of ≤81% and either a high ΔVT2T1 (>27 cm^3^) or a Ki67 of >7%.

**Figure 5:**
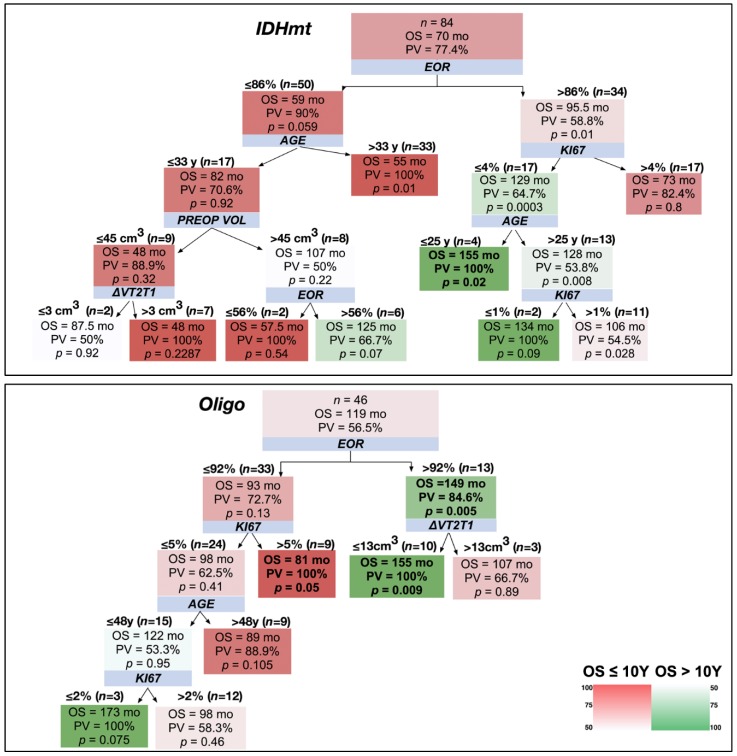
Decision trees applied to the three LGG molecular classes considering 10-year OS as a cut-off. The shading color of the cells indicates the predictive value (PV) of an OS superior (green) or inferior (red) to 10 years (see color heat map). Blue boxes show the variables stratifying the population. On top of the detected subgroups are shown the cut-off values and the number of patients satisfying them. Within the branches, boxes show: the median OS of the patient subgroup, the PV, and the results of the Mann–Whitney test (*p*-value) comparing the subgroup OS with that of the general population (top box). In bold are indicated the subgroups characterized by a PV >75% and a *p* < 0.05.

Considering an OS >10years, none of the astrocytomas IDHwt were long survivors (Figure 5). Conversely, 43.5% of oligodendroglioma patients survived more than 10 years. 84.6% of patients with an EOR of >92% were part of this group and represented about half of the oligodendrogliomas with long survival, the second half being characterized by an age of ≤48 years, although with a PPV of 53.3%. On the other hand, 88.9% of oligodendroglioma patients with an EOR of ≤92% and an age of >48 years died before 10 years. Lastly, 22.6% of IDHmt astrocytoma patients survived >10 years. In these cases, EOR was the major predictor of outcome: if it was ≤86%, it predicted death within 10 years with a PPV of 90%, while if it was >86%, combined with a low proliferation index (Ki67 ≤4%), it predicted long survival with a PPV of 64.7% (Figure 5).

## 3. Discussion

Diffuse grade 2 astrocytomas represent a heterogeneous class of gliomas due to their genetic makeup, clinical behavior, and response to treatment [21]. As a result, patients affected by LGG have a variable outcome, that may be similar, in a patient subset, to that of patients affected by glioblastoma [22]. The treatment paradigm of LGG is based on the principle of the onco-functional balance, which implies maximizing OS together with the preservation of quality of life [23,24]. Therefore, understanding all the possible sources of heterogeneity is essential for a prognostic stratification of patients and useful for programming therapy and setting up appropriate follow-up protocols. Indeed, the definition of guidelines for the management of LGG patients is hampered by the lack of both class II studies and of quantitative approaches that may define the weight of each variable.

For this reason, in the present retrospective monocentric case study including 241 LGG patients, we adopted two statistical approaches: one classical and a novel one based on machine-learning.

We confirmed the reported discrepancies between the 2007 WHO [20] and 2016 WHO [4] classifications of brain tumors. Considering the demographic and clinical features of the different molecular classes, we observed a significant difference in the age of patients affected by oligodendrogliomas, astrocytomas IDHmt, and astrocytomas IDHwt, which is consistent with literature data [21,25]. Most importantly, although the pre-operative volume of gliomas did not differ among the different molecular classes, both EOR and post-operative volumes did. Specifically, the removal of IDHwt lesions was significantly less complete than that of gliomas pertaining to the other molecular classes. Although we observed a significant difference in the anatomical site of occurrence of gliomas, classified in accordance with their molecular class, we excluded that this could be responsible for the differences in EOR. This suggests that EOR could be affected by tumor biological properties other than the preferential location for a specific brain region.

Regarding patient outcome, by employing classical statistical approaches, we confirmed previous studies that observed that EOR is an independent predictor of OS, PFS, and MPFS [8,9,10,11,12,13,14,15,26,27,28,29], also independently from the *IDH* mutational status [26,27]. The molecular class was an independent predictor of OS, PFS, and MPFS also in the present study [5,16,22]. Similarly, KPS and the post-operative volume were predictors of OS and MPFS [16]. We were also able to confirm the prognostic role of the ΔVT2T1 index [9,11,19,30], which is indicative of tumor infiltration [9,11].

It has been recently suggested that a maximal surgical resection may be of the outmost importance in specific molecular classes of LGGs [16]. Therefore, we employed DT as an algorithm to assess how clinical, surgical, histological, and molecular parameters are related to patient outcome and survival. We selected this artificial intelligence approach because it does not require huge amounts of input information and is particularly well suited when dealing with medical data [17,31]. In fact, DT are a white-box model that allows the user to understand the reason behind the choices made by the algorithm; they can deal with both categorical (e.g., sex, molecular class) and numerical data (age) without extensive pre-processing and are robust to missing parameters [17]. Thus, in addition to the classification task, this model was used in this study to identify which parameters are the most informative in relation to the target class and to adopt a quantitative approach, able to define, for each parameter, the best cut-off value to separate classes.

It must be noted that all the parameters identified using DT corresponded to those recognized as independent predictors by the Cox multivariate analyses, further confirming the value of DT. In particular, Table 5 summarizes the major pieces of evidence derived from this machine-learning approach, identifying the features of the patients with an OS of <3 years, >5years, and >10 years, respectively.

When considering the LGG overall, patients with an OS <3 years were those with an EOR <76% and IDHwt, while two groups of patients could reach an OS >10 years: oligodendrogliomas with an EOR of >86% and either a ΔT2T1 index of <40 cm^3^ or second surgery, and astrocytoma IDHmt with an EOR of >86% and a Ki67 of ≤4%. Patients surviving more than 5 years were those with either an EOR of >74%, or those with an EOR of ≤74% but younger than 58 and subjected to second surgery. The favorable influence on outcome of second surgery at relapse, recently suggested by van den Bent’s group [16], seems to be confirmed in our independent case series and extends the indications for second surgery, so far limited to improving the control of the epileptogenic outcome and obtaining a histological assessment of the possible glioma progression [32,33,34,35,36,37,38,39,40,41,42]. A recent meta-analysis of survival outcomes following reoperation in recurrent glioblastoma suggested a possible overestimation of the positive effects of the second surgery on patient outcomes when reoperation is considered a fixed variable [43]. Although our patient population consisted of LGG patients, it would be useful in the future to evaluate the opportunity of exploring the clinical benefits of reoperation, considering this covariate both as time-dependent or fixed.

Furthermore, we observed that distinct factors influence the outcome of patients of the three molecular classes.

In IDHwt, age and Ki67, but not EOR, were the major predictors of outcome; IDHwt patients with a Ki67 of >6% did not survive more than three years, while only those with an age of <28 years and Ki67 of ≤2% survived more than 5 years. This seems to underline the importance of the biological features of IDHwt tumors, as suggested by a recent meta-analysis, indicating that molecular stratification should be ameliorated in order to predict more accurately their outcome [22].

IDHmt patients survived less than 3 years when the EOR was ≤76% and the preoperative volume was >128 cm^3^. Two groups of long-surviving patients were recognized among those with an EOR of >86%: patients younger than 25 with a Ki67 of <4%, and patients older than 25 with a Ki67 of ≤1%. An OS of >5 years was possible in cases with an EOR of >75% and an infiltrative ΔVT2T1 index of ≤40 cm^3^, or in patients with an EOR of ≤ 75% that underwent a second surgery.

Finally, the oligodendroglioma patients with a very short survival were those with an EOR of ≤81% and either a Ki67 of >7% or an infiltrative ΔVT2T1 index of >32 cm^3^. Long-surviving patients had an EOR of >92% and a very low infiltrative index (ΔVT2T1 ≤ 13 cm^3^). Patients that survived more than 5 years presented an EOR of >81% or an EOR of ≤81% but with a Ki67 of <7% and a ΔVT2T1 of <27 cm^3^. In these years, three groups have identified the influence of different levels of EOR on 5-year OS [10,13,14,44]. In the present paper, we confirm the importance of EOR levels and have added additional factors able to further refine prognostic stratification.

## 4. Materials and Methods

The study was approved by the local ethics committee (Consents 102/2011/Sper-196/2014/Em). Written informed consent was obtained from all patients. Clinical investigations were conducted according to the principles expressed in the Declaration of Helsinki.

### 4.1. Patients Included in the Study

Adult patients (age >18 years) who underwent surgical resection for a symptomatic, newly diagnosed glioma, WHO grade II (LGG), at the Neurosurgery Department of the Hospital of Udine between 2000 and 2016 were enrolled; none had been previously subjected to chemo- and/or radiotherapy. Extensive surgical resection was carried out at diagnosis. Radio- and/or chemotherapy as well as second surgery was carried out in cases with tumor progression. For each patient, the following data had to be available: age, gender, Karnofsky performace status (KPS), molecular classification according to WHO 2016, Ki67 expression, pre-and post-operative tumor volume, extent of resection (EOR), and preoperative infiltration index expressed by the ΔVT2T1 value, as evaluated by magnetic resonance imaging (MRI, see below). Patients presented a follow-up of at least 12 months. The following data were also evaluated, when possible: the methylation status of the MGMT promoter, ATRX and p53 expression.

### 4.2. Histological and Molecular Examination

Histological examination, immunohistochemistry (IHC) for Ki67, p53, and ATRX (Supplementary Figure S3), evaluation of 1p/19q co-deletion by FISH and assessment of *IDH*1/2 gene mutations, and of the methylation status of the MGMT promoter were performed as in [19,30].

### 4.3. Volumetric Analysis

Pre- and post-operative tumor volume, extent of resection (EOR), and preoperative ΔVT2T1 value were calculated as previously [9,11]. Specifically, pre- and post-operative tumoral segmentations were performed manually across all MRI slices with the OSIRIX software tool 3.0 on the basis of T2 axial slices by T.I. (10 years of experience) and M.S. (10 years of experience). The EOR was measured on T2-weighted MRI axial images as follows: ((pre-operative tumor volume — post-operative tumor volume) / pre-operative tumor volume). The pattern of tumor growth was assessed by analyzing the infiltration index ΔVT2T1, defined as follows: (preoperative volumetric tumor volume segmented on T2-weighted MRI images — preoperative volumetric tumor volume segmented on T1-weighted images).

### 4.4. Statistical Analysis

Characteristics of the study population are described using means ± s.d. or median and range for continuous variables and percentages for categorical variables. Data were tested for normal distribution using the Shapiro–Wilk test. One-way Anova followed by Bonferroni post-test or Kruskall–Wallis test, followed by Dunn’s post-test were used to compare continuous variables among groups. OS, PFS, and MPFS were defined as the time between initial surgery and, respectively, death (OS); demonstration of an increase in tumor size on follow-up imaging, malignant progression, and/or death (PFS); demonstration of gadolinium enhancement on follow-up imaging and/or higher-grade tumor on subsequent biopsy or death (MPFS). OS, PFS, and MPFS were described using the Kaplan–Meier approach. Log rank test was used to compare survival curves. Survival was analyzed by Cox proportional hazard models, following verification of the assumptions based on scaled Schoenfeld residuals [45]. Covariates with *p* < 0.05 at univariable analysis were selected for multivariable stepwise analysis. Analyses were conducted with Stata/SE 16.0 for Mac software and R software environment for statistical calculation.

The analysis with the DT was carried out using Orange Canvas, a user-interface for machine learning methods based on the scikit-learn Python library. This library uses an optimized version of the CART (classification and regression trees) algorithm [44], that selects features and cut-offs yielding the largest information gain at each node. In order to avoid the creation of excessively complex trees, that could have overfitted the data, the decision trees were limited to a maximum depth of 6 nodes including less than 5 patients and were not split any further; the minimum number of patients in leaves was 2. Every node was set up in order to offer the following information relative to each subgroup: the majority class of the node, including either “patients above threshold” or “patients below threshold”; the percentage of patients corresponding to the majority class, i.e., the predictive value that indicates the pureness of the node; the *p*-value resulting from the Mann–Whitney test to evaluate the distribution difference between the initial population and the node population. A *p*-value below 0.05 was considered significant. For random forest and ADA boost, it was used the default initial configuration provided by Orange.

## 5. Conclusions

A DT approach was applied to a large monocentric series of 241 patients with a diagnosis of LGG in order to stratify them prognostically using a multiparametric approach. The main features of this algorithm consist of a hierarchical stratification of prognostic factors that are able to uncover which, among the different parameters evaluated, are the most relevant ones, and the identification, for each prognostic factor, of cutoff values that are able to identify patients that are at particular risk. This approach, although it necessitates being validated in independent patient cohorts, can become useful support in patient management.

## Figures and Tables

**Table 1:** Clinical and neuroradiological features of the 241 symptomatic LGG patients included in the study. For the features, tumor side, tumor location, post-operative volume, and extent of resection are reported both the %, within the molecular class, of the different feature subgroups, and the percentage of cases pertaining to each molecular class within each feature subgroups. *^,^** *p* < 0.05 vs. astrocytoma, IDH wildtype and astrocytoma, IDH mutant, respectively. Boldfacing represents statistical significance (*p* < 0.05) from Dunn’s post-test.

Clinical Feature	All Cases *n* = 241 (100%)		Astrocytoma, IDH Wildtype *n* = 27 (11.2%)			Astrocytoma, IDH Mutant *n* = 137 (56.8%)			Oligodendroglioma *n* = 77 (32.0%)			
	N (%)	Median (range)	N (%) by Class	% by Feature	Median (range)	N (%) by Class	% by Feature	Median (range)	N (%) by Class	% by Feature	Median (range)	*p*
Sex												0.766
Male	144 (59.8)		18 (66.7)			81 (59.1)			45 (58.4)			
Female	97 (40.2)		9 (33.3)			56 (40.9)			32 (41.6)			
**Age at Surgery (years)**		39 (19–75)			51 (23–75)			36 (19–74) *			41 (22–66) **	**0.0001**
**Karnofsky Performance Status**		100 (80–100)			100 (80–100)			100 (80–100)			100 (80–100)	0.866
**Tumor side**												0.935
Left	129 (53.5)		15 (55.6)	11.6		74 (54.0)	57.4		40 (51.9)	31.0		
Right	112 (46.5)		12 (44.4)	10.7		63 (46.0)	56.3		37 (48.1)	33.0		
**Tumor location**												**0.006**
Frontal lobe	97 (40.2)		5 (18.5)	5.2		58 (42.3)	59.8		34 (42.2)	35.0		
Insular lobe	72 (29.2)		9 (33.3)	12.5		48 (35.0)	66.7		15 (19.5)	20.8		
Parietal lobe	33 (13.7)		4 (14.8)	12.1		18 (13.1)	54.5		11 (14.3)	33.3		
Temporal lobe	39 (16.2)		9 (33.3)	23.1		13 (9.5)	33.3		17 (22.1)	43.6		
**Pre-operative volume (cm^3^)**		44 (6–260)			44 (6–133)			45 (6–260)			40 (8–159)	0.1936
**ΔVT2T1 index (cm^3^)**		13 (0–95)			18 (0–68)			14 (0–95)			11 (0–66)	0.1841
**Post-operative volume (cm^3^)**		7 (0–125)			11 (0–57)			7 (0–125)			6 (0–45) *, **	**0.027**
<10 cm^3^	143 (59.3)		11 (4.7)	7.7		81 (59.1)	56.6		51 (66.2)	35.7		
10–19 cm^3^	51 (21.2)		8 (29.6)	15.7		25 (18.2)	49.0		18 (23.4)	35.3		
20–29 cm^3^	22 (9.4)		1 (3.7)	4.5		14 (10.2)	63.6		7 (9.1)	31.8		
>30 cm^3^	25 (10.4)		7 (25.9)	28.0		17 (12.4)	68.0		1 (1.3)	4.0		
**Extent of Resection (%)**		86 (28–100)			69 (34–100)			86 (28–100)			87 (50–100)*	**0.008**
≥90%	99 (41.1)		9 (33.3)	9.1		54 (39.4)	54.5		36 (46.8)	36.4		
70–89%	97 (40.2)		4 (14.8)	4.1		60 (43.8)	61.9		33 (42.9)	34.0		
<70%	45 (18.7)		14 (51.9)	31.1		23 (16.8)	51.1		8 (10.4)	17.8		

**Table 2:** Pathological and molecular features of the 241 symptomatic LGG patients included in the study. For the feature, WHO 2007 LGG type are reported in the %, within the molecular class, of the different WHO 2007 types as well as the percentage of cases pertaining to each molecular class within each WHO 2007 type. *, ** *p* < 0.05 vs. astrocytoma, IDH wildtype and astrocytoma, IDH mutant, respectively. Boldfacing represents statistical significance (*p* < 0.05) from Dunn’s post-test.

Pathological Feature	All Cases *n* = 241 (100%)		Astrocytoma, IDH Wildtype *n* = 27 (11.2%)			Astrocytoma, IDH Mutant *n* = 137 (56.8%)			Oligodendroglioma *n* = 77 (32.0%)			
	N (%)	Median (range)	N (%) by Class	% by Category	Median (range)	N (%) by Class	% by Category	Median (range)	N (%) by Class	% by Category	Median (range)	*p*
**WHO 2007 LGG type**												**0.0001**
Astrocytoma	151 (62.7)		25 (92.6)	16.6		115 (83.9)	76.2		11 (14.3)	7.3		
Oligodendroglioma	25 (10.4)		0	0		0	0		25 (32.5)	100		
Oligoastrocytoma	65 (27.0)		2 (7.4)	3.1		22 (16.1)	33.8		41 (53.2)	63.1		
**Ki67 expression (%)**		4 (1–22)			3 (1–22)			5 (1–15)			4 (1–12)	0.284
**Number of Mitoses/10 HPF**		1 (0–10)			1 (0–6)			1 (0–10)			1 (0–8)	0.529
**P53 expression** (%) (*n* = 232)	160 (69.0)		12 (46.2)			123	90.4		25	35.7		**<0.0001**
**ATRX down-regulation** (*n* = 203)	116 (57.1)		3 (18.8)			105	91.3		8	11.1		**<0.0001**
***IDH*** **1 / *IDH*2 mutation**	213 (88.8)		0			137	100 *		76	100 *		**<0.0001**
**1p/19q co-deletion** (*n* = 238)	77 (32.3)		1 (4)			0	0		76	100		**<0.0001**
**MGMT promoter methylation** (*n* = 231)	202 (87.5)		15 (60)			115	86.5		72	98.6		**<0.0001**
**Average MGMT promoter Methylation** (%) (*n* = 195)		25.5 (2.5–80.75)			14.3 (3.2–43.7)			24.1 (3.2–64.7) *			32.4 (2.5–80.7) *, **	**0.0001**

**Table 3:** Univariate analysis of clinical, histological and molecular parameters in relation to overall survival (OS), progression-free survival (PFS), and malignant progression-free survival (MPFS) in 241 patients with low-grade-gliomas.

Clinicopathological Feature	Reference Variable	OS			PFS			MPFS		
		HR	95% CI	*p*	HR	95% CI	*p*	HR	95% CI	*p*
Age*		1.022	1.007–1.037	**0.004**	1.006	0.994–1.019	0.334	1.019	1.005–1.033	0.006
**Sex**	Female	0.988	0.688–1.420	0.951	1.139	0.844–1.537	0.395	0.964	0.695–1.336	0.824
**KPS** *		0.953	0.924–0.982	**0.002**	0.979	0.952–1.007	0.144	0.955	0.928–0.982	0.001
**Tumor site**	Left hemisphere	0.760	0.533–1.083	0.128	0.680	0.509–0.910	**0.01**	0.741	0.538–1.019	0.065
Pre–operative volume *,#		3.288	1.874–5.766	**<0.0001**	2.217	1.394–3.524	**0.001**	3.872	2.305–6.503	**<0.0001**
Infiltrative growth index (ΔVT2T1) *		1.034	1.025–1.044	**<0.0001**	1.035	1.025–1.045	**<0.0001**	1.036	1.027–1.044	**<0.0001**
Post-operative volume *		1.015	1.009–1.021	**<0.0001**	1.021	1.015–1.027	**<0.0001**	1.015	1.010–1.021	**<0.0001**
% EOR *		0.954	0.943–0.964	**<0.0001**	0.961	0.952–0.970	**<0.0001**	0.956	0.946–0.966	**<0.0001**
**WHO 2007 LGG type**	Astrocytoma									
Oligodendroglioma		0.255	0.117–0.554	**0.001**	0.441	0.259–0.751	**0.003**	0.417	0.233–0.748	**0.003**
Oligoastrocytoma		0.637	0.425–0.955	**0.029**	0.641	0.459–0.895	**0.009**	0.624	0.431–0.902	**0.012**
% Ki67*		1.084	1.016–1.157	**0.014**	1.059	1.004–1.117	**0.033**	1.051	0.990–1.115	0.102
Number of Mitosis 10HPF *		1.025	0.913–1.150	0.677	1.053	0.958–1.158	0.283	1.019	0.918–1.131	0.722
***IDH*1 or *IDH*2** **mutation**	**No**	0.189	0.111–0.322	**<0.0001**	0.368	0.233–0.579	**<0.0001**	0.259	0.158–0.422	**<0.0001**
**Chromosome 1p/19q** **co–deletion**	**No**	0.463	0.308–0.696	**<0.0001**	0.591	0.431–0.810	**0.001**	0.529	0.370–0.754	**<0.0001**
**Molecular class**	IDH wild type									
IDH mutant		0.236	0.137–0.405	**<0.0001**	0.434	0.272–0.691	**<0.0001**	0.313	0.190–0.516	**<0.0001**
IDH mutant and 1p/19q codeletion		0.117	0.063–0.216	**<0.0001**	0.269	0.163–0.448	**<0.0001**	0.177	0.101–0.308	**<0.0001**
P53 expression *		1.094	0.735–1.629	0.658	1.281	0.924–1.775	0.137	1.048	0.736–1.492	0.796
**ATRX downregulation**	**Yes**	1.039	0.694–1.557	0.850	0.939	0.679–1.296	0.701	1.089	0.766–1.548	0.634
**MGMT promoter methylation**	**No**	0.497	0.292–0.847	**0.010**	0.447	0.286–0.699	**<0.0001**	0.571	0.351–0.927	**0.023**
**Radiotherapy**	**No**	5.596	2.459–12.732	**<0.0001**	1.926	1.295–2.864	**0.001**	5.115	2.690–9.725	**<0.0001**
**Chemotherapy**	**No**	1.876	1.107–3.179	**0.019**	1.916	1.288–2.852	**0.001**	2.365	1.458–3.837	**<0.0001**
**Second surgery**	**No**	0.522	0.360–0.757	**0.001**	1.220	0.913–1.632	0.179	0.899	0.651–1.242	0.518

HR = hazard ratio; CI = confidence interval; EOR = extent of surgical resection. * modeled as continuous variable. # Log-transformed variable. Boldfacing represents statistical significance (*p* < 0.05) from two-sided tests (Cox regression).

**Table 4:** Multivariate analysis of clinical, histological and molecular parameters in relation to overall survival (OS), progression-free survival (PFS), and malignant progression-free survival (MPFS) in 241 patients with low-grade-glioma.

		OS			PFS			MPFS		
	Reference Variable	HR	95% CI	*p*	HR	95% CI	*p*	HR	95% CI	*p*
**KPS** *		0.948	0.913–0.984	**0.006**				**0.954**	**0.922–0.988**	**0.008**
Pre–operative volume*#								**3.010**	**1.428–6.345**	**0.004**
Infiltrative growth index (ΔVT2T1)*					**1.024**	**1.006–1.041**	**0.008**	**1.019**	**1.002–1.036**	**0.024**
Post–operative volume *		0.980	0.962–0.998	**0.028**				**0.980**	**0.964–0.996**	**0.016**
% EOR *		0.952	0.933–0.972	**<0.0001**	**0.971**	**0.956–0.986**	**<0.0001**	**0.957**	**0.940–0.974**	**<0.0001**
% Ki67 *		1.075	1.013–1.142	**0.018**						
**Molecular class**	IDH wild type									
IDH1/2 mutant		0.374	0.187–0.749	**0.005**	**0.409**	**0.237–0.706**	**0.001**	**0.316**	**0.169–0.593**	**<0.0001**
IDH mutant and 1p/19q codeletion		0.179	0.083–0.388	**<0.0001**	**0.290**	**0.159–0.527**	**<0.0001**	**0.175**	**0.088–0.346**	**<0.0001**
**Second Surgery**	No	0.644	0.423–0.979	**0.039**						

HR = hazard ratio; CI = confidence interval; EOR = extent of surgical resection. * modeled as continuous variable. # Log-transformed variable. Boldfacing represents statistical significance (*p* < 0.05) from two-sided tests (Cox regression).

**Table 5:** Schematic representation of the principal features, derived from the decision tree analysis, that characterize homogeneous groups of patients in terms of prediction of OS <3, >5, and >10 years, respectively. In each cell are indicated the features of the subgroup and the predictive value (PV). The analyses were conducted on the whole cohort of patients (see Figure 2) and separately on the three molecular classes (see Figure 3): astrocytomas, IDH wildtype (IDHwt); astrocytomas, IDH mutant (IDHmt); oligodendrogliomas, IDH mutant and 1p/19q co-deleted (oligodendroglioma).

**All Patients**		
**OS <3 Years**	**OS >5 Years**	**OS >10 Years**
EOR ≤ 76%	EOR >74% **PV = 83.8%**	EOR >86%
IDHwt **PV *=* 78.6%**		Oligodendroglioma
		ΔVT2T1 ≤ 10 cm^3^ or 2^nd^ surgery **PV = 100%** *(if* ΔVT2T1 ≤ 10 cm^3^); **71.4%** (if 2^nd^ surgery)
	EOR ≤74%	EOR >86%
	Age ≤58 years	IDHmt
	2^nd^ surgery **PV *=* 58.3%**	Ki67 ≤ 4% **PV = 64.7%**
**IDHwt**		
**OS <3 years**	**OS >5 years**	**OS >10 years**
Ki67 >6% **PV = 100%**	Ki67 ≤2%	–
	Age ≤58 years **PV = 100%**	
**IDHmt**		
**OS < 3 years**	**OS > 5 years**	**OS > 10 years**
EOR ≤76%	EOR >75%	EOR >86%
Pre-operative volume >128 cm^3^ **PV = 66.7%**	ΔVT2T1 ≤40 cm^3^ **PV = 84.2%**	Age ≤25 years
		Ki67 ≤4% ***PV* = 100%**
	EOR ≤75%	EOR >86%
	2^nd^ surgery **PV = 75%**	Age >25 years
		Ki67<1% **PV = 100%**
**Oligodendroglioma**		
EOR ≤81%	EOR >81% **PV = 95.8%**	EOR >92%
Ki67 >7% or ΔVT2T1 >32 cm^3^ **PV = 100%** (if Ki67 >7%); **71.4%** *(if* ΔVT2T1 > 32 cm^3^)		ΔVT2T1 ≤13 cm^3^ **PV = 100%**
EOR ≤81%		
ΔVT2T1 Ki67 ≤7% **PV = 88.9%**		

**Table 6:** Classification accuracy resulting from leave-one-out evaluation using three machine learning approaches based on decision trees. The results are divided by the three OS threshold considered.

Decision Tree Version	OS >3 years	OS >5 years	OS >10 years
**Decision Tree**	0.799	0.777	0.716
**Random Forest**	0.841	0.782	0.777
**ADA Boost**	0.785	0.686	0.676

## Supplementary Materials

The following are available online at https://www.mdpi.com/2072-6694/12/1/50/s1, Figure S1: Decision trees of LGG with 3-year, 5-year and 10-year PFS; Figure S2: Decision trees of LGG with 3-year, 5-year and 10-year MPFS; Figure S3: Immunohistochemistry.
