# Supplementary material for: Application of an Artificial Intelligence Algorithm to Prognostically Stratify Grade II Gliomas
Source: Cancers (Basel). 2019 Dec 22;12(1):50. doi: 10.3390/cancers12010050 (PMC7016715; doi:10.3390/cancers12010050)
Supplement: Supplementary file 1 [file cancers-12-00050-s001.zip › Supplementary figures.docx]

**Supplementary figures**

**Supplementary Figure 1: Decision trees of LGG with 3-year, 5-year and 10-year PFS.** On one top corner of each box we have indicated the cut-off value defining the patients comprised within the box. Within the box we have indicated whether the median OS of the patient subset was lower or higher than 3 **(A)**, 5 **(B)** or 10 **(C)** years. Moreover, the positive predictive values (PPV) and the fraction of patients satisfying the cutoff criteria are shown. The color of the cells indicates the PPV in predicting an OS superior (green scale) or inferior (red scale) to 3 **(A)**, 5 **(B)** or 10 **(C)** years.

**Supplementary Figure 2: Decision trees of LGG with 3-year, 5-year and 10-year MPFS.** On one top corner of each box we have indicated the cut-off value defining the patients comprised within the box. Within the box we have indicated whether the median OS of the patient subset was lower or higher than 3 **(A)**, 5 **(B)** or 10 **(C)** years. Moreover, the positive predictive values (PPV) and the fraction of patients satisfying the cutoff criteria are shown. The color of the cells indicates the PPV in predicting an OS superior (green scale) or inferior (red scale) to 3 **(A)**, 5 **(B)** or 10 **(C)** years.

**Supplementary Figure 3: Immunohistochemistry.** Representative pictures of immunohistochemical detection of IDH1-R132H, ATRX, and Ki-67. In the left column are reported examples of samples positive for the assayed markers, while in the right column are shown tumors not expressing the tested proteins (pictures obtained at 40X magnification).
