# Supplementary figures and images for: Application of an Artificial Intelligence Algorithm to Prognostically Stratify Grade II Gliomas
Source: Cancers (Basel). 2019 Dec 22;12(1):50. doi: 10.3390/cancers12010050 (PMC7016715; doi:10.3390/cancers12010050)

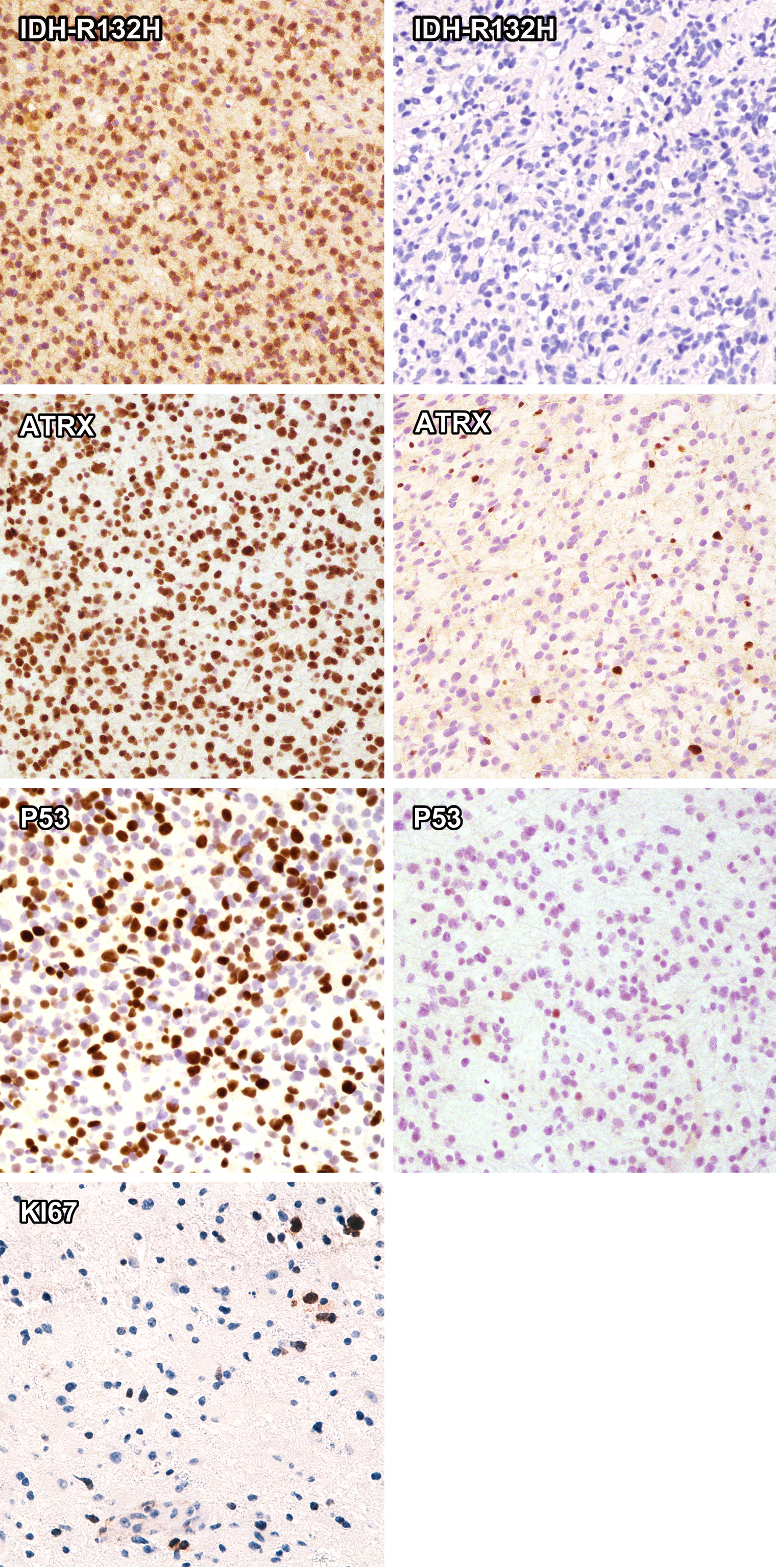

Supplement: Supplementary file 1 [file cancers-12-00050-s001.zip › Supplementary Figure 3.tif]

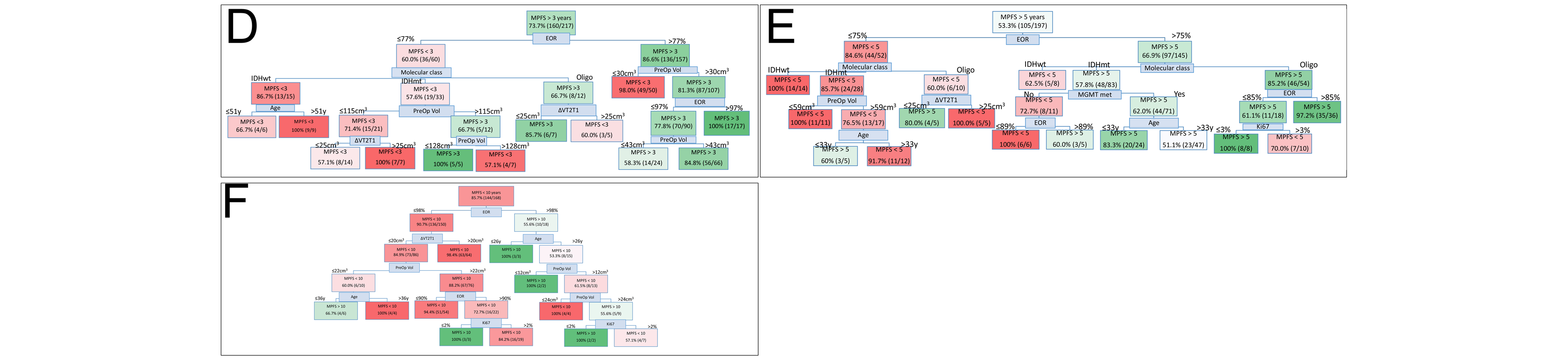

Supplement: Supplementary file 1 [file cancers-12-00050-s001.zip › Supplementary Figure 2.tif]

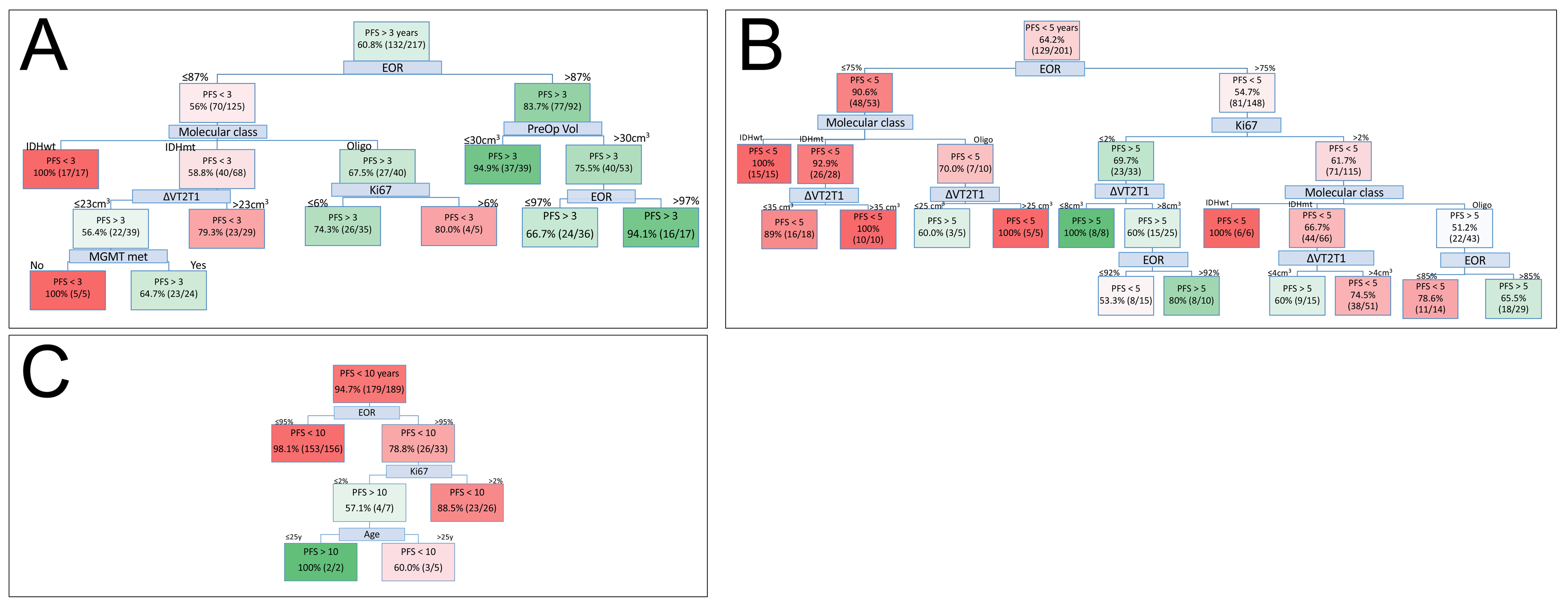

Supplement: Supplementary file 1 [file cancers-12-00050-s001.zip › Supplementary Figure 1 .tif]
